# Draft genome sequences of five *Calonectria* species from *Eucalyptus* plantations in China, *Celoporthe dispersa*, *Sporothrix phasma* and *Alectoria sarmentosa*

**DOI:** 10.1186/s43008-019-0023-5

**Published:** 2019-12-27

**Authors:** Feifei Liu, Shuaifei Chen, Maria A. Ferreira, Runlei Chang, Mohammad Sayari, Aquillah M. Kanzi, Brenda D. Wingfield, Michael J. Wingfield, David Pizarro, Ana Crespo, Pradeep K. Divakar, Z. Wilhelm de Beer, Tuan A. Duong

**Affiliations:** 1 State Key Laboratory of Tree Genetics and Breeding (SKLTGB), Chinese Academy of Forestry (CAF), Haidian District, Beijing, 100091 China; 2 China Eucalypt Research Centre (CERC), Chinese Academy of Forestry (CAF), ZhanJiang, 524022 GuangDong Province China; 3 Department of Biochemistry, Genetics and Microbiology (BGM), Forestry and Agricultural Biotechnology Institute (FABI), University of Pretoria, Pretoria, 0028 South Africa; 4 Department of Plant Pathology, Universidade Federal de Lavras (Federal University of Lavras), Postal Box 3037, Lavras, 37200-000 Brazil; 5 Departamento de Farmacología, Farmacognosia y Botánica, Facultad de Farmacia, Universidad Complutense de Madrid, Plaza de Ramón y Cajal s/n, 28040 Madrid, Spain

**Keywords:** *Alectoria sarmentosa*, *Calonectria* species, *Celoporthe dispersa*, Eucalyptus leaf disease, Fungal pathogens, *Sporothrix phasma*

## Abstract

Draft genome sequences of five *Calonectria* species [including *Calonectria aciculata*, *C. crousiana*, *C. fujianensis*, *C. honghensis* and *C. pseudoturangicola*], *Celoporthe dispersa*, *Sporothrix phasma* and *Alectoria sarmentosa* are presented. Species of *Calonectria* are the causal agents of Eucalyptus leaf blight disease, threatening the growth and sustainability of *Eucalyptus* plantations in China. *Celoporthe dispersa* is the causal agent of stem canker in native *Syzygium cordatum* and exotic *Tibouchina granulosa* in South Africa. *Sporothrix phasma* was first discovered in the infructescences of *Protea laurifolia* and *Protea neriifolia* in South Africa. *Alectoria sarmentosa* is fruticose lichen belongs to the alectorioid clade of the family Parmeliaceae. The availability of these genome sequences will facilitate future studies on the systematics, population genetics, and genomics of these fungi.

## IMA GENOME-F 12A

**Draft genome sequences of five** ***Calonectria*** **species from** ***Eucalyptus*** **plantations in China.**

### Introduction

Species in the genus *Calonectria* have a global distribution, particularly in tropical and subtropical regions of the world (Lombard et al. 2010c). These fungi include numerous important plant pathogens causing diseases on shoots, leave and roots of agricultural and forestry crops, which have led to significant economic impacts worldwide (Lombard et al. 2010c). The disease symptoms include cutting rot, damping off, leaf spots, leaf blight, shoot blight, defoliation as well as stem cankers and fruit rot (Crous 2002).

In the past 10 years, due to the influence of the phylogenetic species concept, many novel species of *Calonectria* have been described (Lombard et al. 2010a; Lombard et al. 2010b; Chen et al. 2011b; Xu et al. 2012; Alfenas et al. 2013a; Alfenas et al. 2013b; Lombard et al. 2016; Li et al. 2017; Liu and Chen 2017; Pham et al. 2019). This genus currently includes 171 recognized species residing in 10 different species complexes. Among these, 34 species, belonging to *C. candelabra*, *C. cohounii*, *C. cylindrospora*, *C. kyotensis* and *C. reteaudii* species complexes, were first discovered and described from China (Lombard et al. 2016; Li et al. 2017; Liu and Chen 2017; Pham et al. 2019). In 2015, a survey conducted in a relatively small area in southern China led to the discovery of 18 novel species from soil and symptomatic *Eucalyptus* plant tissues (Chen et al. 2011b; Lombard et al. 2015). This has highlighted the rich species diversity of *Calonectria* in China.

Despite their economic importance for *Eucalyptus* plantation forestry in China and other parts of the world, little is known regarding the biology and genetic determinants of virulence in *Calonectria* species. In this study, we sequenced the genomes of five important *Calonectria* species described from China. The overall aim was to facilitate future research regarding these important fungi, especially relating to their taxonomy, population genetics, and pathogenicity.

### Sequenced strains

*Calonectria aciculata*: China: *YunNan*: isol. leaves of an *E. urophylla* × *E. grandis* hybrid clone, 16 Nov. 2014, *S.F. Chen & J.Q. Li* (PREM 61941 – holotype; CMW 47645 = CERC 5342 = CBS 142883 – ex-type culture).

*Calonectria crousiana*: China: *FuJian*: isol. leaves of *Eucalyptus grandis*, Aug. 2007 *M.J. Wingfield* (PREM 60453 – holotype; CMW 27249 = CBS 127198 – ex-type culture).

*Calonectria fujianensis*: China: *FuJian*: isol. leaves of *Eucalyptus grandis*, Aug. 2007 *M.J. Wingfield* (PREM 60460 – holotype; CMW 27257 = CBS 127201 – ex-type culture).

*Calonectria honghensis*: China: *YunNan*: isol. soil collected in a *Eucalyptus* plantation, 14 Nov. 2014, *S.F. Chen & J.Q. Li* (PREM 61943 – holotype; CMW 47669 = CERC 5572 = CBS 142885 – ex-type culture).

*Calonectria pseudoturangicola*: China: *FuJian*: isol. soil collected in the campus of Fujian Agriculture and Forestry University (FAFU), 14 Dec. 2014, *S.F. Chen* (PREM 61948 – holotype; CMW 47496 = CERC 7126 = CBS 142890 – ex-type culture).

### Nucleotide sequence accession numbers

The draft genome data for the five *Calonectria* isolates have been deposited at DDBJ/EMBL/GenBank under BioProject PRJ562676. The accession numbers for each of the species are presented in Table 1.

**Table 1:** Statistics of *Calonectria* genomes sequenced in this study

Species	Isolate number	Accession number	Total Bases	Read Count	GC (%)	Scaffold number	Assembly size (Mb)	N50 (bp)	L50	Coverage	Complete BUSCO (%)	Predicted gene models	Gene density (ORFs/Mb)
*C. aciculata*	CMW 47645	VTGE01000000	2,442,674,772	9,731,772	47.721	221	61.6	675,696	25	39.7	98.7	15,556	252
*C. crousiana*	CMW 27249	VTGD01000000	6,593,014,992	26,266,992	48.718	358	58.1	419,924	46	113.5	98.5	14,967	257
*C. fujianensis*	CMW 27257	VTGC01000000	4,205,231,410	16,753,910	46.805	194	61.5	695,013	24	68.4	98.8	15,489	251
*C. honghensis*	CMW 47669	VTGB01000000	4,685,120,820	18,665,820	47.363	141	61.7	1,034,491	19	75.9	98.8	15,640	253
*C. pseudoturangicola*	CMW 47496	VTGA01000000	4,871,214,228	19,407,228	47.666	155	62.1	875,460	22	78.5	98.7	14,183	228

### Materials and methods

Genomic DNA was extracted from single conidial cultures grown on malt yeast broth (2% malt extract, 0.5% yeast extract) using the method described by Duong et al. (2013). To verify the identification of all the sequenced isolates, PCR amplification and sequencing of the partial elongation factor gene (*tef1*) for the extracted DNA was carried out on each isolate. The *tef1* sequences were then aligned against the sequences which developed in previous studies (Chen et al. 2011b; Li et al. 2017). After the identification, a phylogenetic tree reflecting the position of these five species in relation to other *Calonectria* species was subsequently produced based on the four gene regions (*cmdA*, *his3*, *tef1* and *but2*). The sequences of representative isolates of the different species in this genus were obtained from GenBank, as reported by Liu and Chen (2017), and aligned using MAFFT version 7 (https://mafft.cbrc.jp/alignment/server/) (Katoh and Standley 2013). Phylogenetic analysis using maximum likelihood (ML), was conducted with PhyML v. 3.1 (Model = TIM2 + G; Guindon and Gascuel 2003). Confidence levels for the nodes were determined using 1000 bootstrap replicates. Final consensus trees were viewed and edited in MEGA 7.

The genomic DNA was submitted to Macrogen (South Korea), where one pair-end library with 550 bp median insert size was prepared using TruSeq DNA PCR-free protocol, and sequenced on Illumina Hiseq 2500 platform to get 250 bp pair-end reads. The quality of the data obtained was assessed using the software FastQC v. 0.11.5 (Afgan et al. 2016). Poor quality data and adapters were removed using the program Trimmomatic v. 0.36 (Bolger et al. 2014).

*De novo* assembly of the genome was carried out with SPAdes v. 3.9 (Bankevich et al. 2012) using trimmed pair-end data. Contigs that were smaller than 500 bp or with less than 20% of average K-mer coverage were removed from the assemblies. The filtered contigs were further placed into scaffolds with SSPACE-standard v. 3.0 (Boetzer et al. 2011) using the information from pair-end reads. Assembly gaps were filled or extended using GapFiller v. 1.10 (Boetzer and Pirovano 2012) with the paired-end data. Final assemblies were subjected to completeness assessment using the program Benchmarking Universal Single-Copy Orthologs (BUSCO) v. 2.0 (Simão et al. 2015) utilizing the dataset for Sordariomycetes. The program AUGUSTUS v. 3.2.2 was used to estimate the number of protein coding genes encoded by these genomes utilizing the species model for *Magnaporthe grisea* (Stanke et al. 2006).

### Results and discussion

**Fig. 1:**
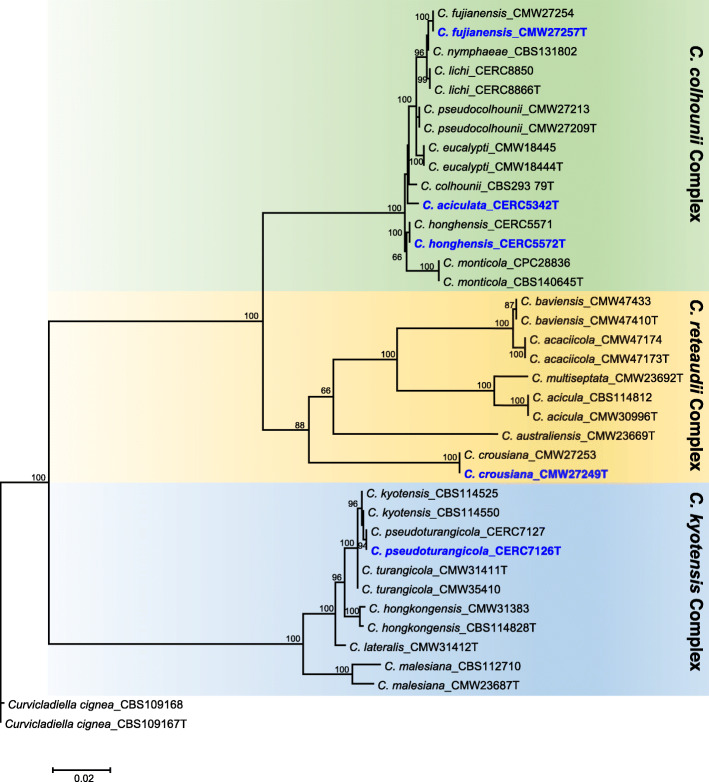
Maximum likelihood tree based on partial gene sequences of *cmdA*, *his3*, *tef1* and *but2* gene sequences (Chen et al. 2011b; Li et al. 2017; Liu and Chen 2017; Pham et al. 2019). Bootstrap values ≥65% are shown. The isolates used in this study are indicated in blue and bold

The *tef1* gene from the PCR products confirmed the five *Calonectria* species, and a phylogenetic tree based on the four gene regions (*cmdA*, *his3*, *tef1* and *but2*) reflecting the position of these five species in relation to other *Calonectria* species was produced (Fig. 1). The genomes of *C. aciculata*, *C. crousiana*, *C. fujianensis*, *C. honghensis* and *C. pseudoturangicola* were subsequently sequenced and assembled. Paired-end sequences of the libraries for the five isolates yielded from 9.7 to 26.2 million reads per library. These draft assemblies had scaffolds ranging from 141 to 358 in number. The assembled genome sizes were 58.1 Mb to 62.1 Mb in size. The N50 of the assemblies ranged from 419.9 Kb to 1034.5 Kb. The assemblies had BUSCO completeness scores ranging from 98.5 to 98.8%. The number of gene models ranged from 14,183 to 15,640. Statistics for all assembled genomes are presented in Table 1.

The statistics for the five *Calonectria* genomes were comparable to that of the draft genome sequence for *C. pseudoreteaudii* strain YA51, published by Ye et al. (2018), which had 507 scaffolds, 14,355 gene models, a total assembly size of 63.57 Mb, and a N50 of 1320 Kb. The availability of the genome sequences for the five *Calonectria* species presented in this study will enable comparative genomics studies to be conducted for these and various other species in the genus. They will also facilitate future investigations regarding the factors involved in pathogenicity, mating behaviour, and evolution of these important plant pathogens.

*Authors:* **F.F. Liu, S.F. Chen** * **, B.D. Wingfield, M.J. Wingfield, T.A. Duong.**

*Contact: shuaifei.chen@gmail.com

## IMA GENOME-F 12B

**Draft genome sequences of** ***Celoporthe dispersa*** **, a causal agent of canker diseases.**

### Introduction

The Cryphonectriaceae family includes several phytopathogens, and many of these pathogens cause severe damage to commercial plantations, especially *Eucalyptus* spp. (Chen et al. 2011a; Soares et al. 2018; Wang et al. 2018a). *Celoporthe dispersa* was first reported causing stem canker in native *Syzygium cordatum* and exotic *Tibouchina granulosa* in South Africa (Nakabonge et al. 2006). Pathogenicity trials conducted on *E. grandis* and *T. granulosa* showed that *C. dispersa* is pathogenic on both hosts, highlighting that *C. dispersa* could threaten commercial grown *Eucalyptus* trees in South Africa (Nakabonge et al. 2006). Within the Cryphonectriacea family, sequenced genomes are available for four species, including *Chrysoporthe cubensis*, *Chr. deuterocubensis*, *Chr. austroaficana* (Wingfield et al. 2015a; Wingfield et al. 2015b), and *Cryphonectria parasitica* (http://genome.jgi.doe.gov/Crypa2/Crypa2.info.html). The sequencing of this additional *C. dispersa* genome will be valuable for comparative genome studies within the Cryphonectriaceae family, and for improving disease management strategies, as well as preventing the threat to commercial plantations.

### Sequenced strains

*Celoporthe dispersa*: South Africa, Limpopo Province, Tzaneen, *Syzygium cordatum*, 2003, M. Gryzenhout (CMW 9976 = CBS 118782, PREM 58897 – dried culture).

### Nucleotide sequence accession numbers

The genome sequence of *Celoporthe dispersa* (isolate number CMW 9976) has been deposited in DDBJ/EMBL/GenBank databases under the accession number WAID00000000. The version described in this paper is WAID00000000.

### Material and methods

Genomic DNA was extracted from freeze-dried mycelium of isolate CMW 9976 grown in malt yeast broth (2% Malt extract, 0.5% yeast extract; Biolab, Midrand, South Africa) using the Qiagen® Genomic-tip DNA extraction protocol for plants and fungi. Nanopore sequencing was conducted using the MinION sequencing device. The sequencing library was prepared using the Genomic DNA by Ligation (SQK-LSK109) protocol. The library was loaded on a MinION flowcell (R9.5.1) and sequencing was run for 48 h. Base calling was conducted using ONT Guppy basecalling software v 2.3.7.

Nanopore reads were error-corrected using Canu v 1.8 (Koren et al. 2017). The genome was assembled using smartdenovo (Istace et al. 2017), with corrected reads from Canu as input. The assembly was polished using base level signal from the ONT raw reads using the program Nanopolish (Jain et al. 2018). The program AUGUSTUS (Stanke and Morgenstern 2005) was used for prediction of protein coding genes present in *C. dispersa* genome. The *Fusarium graminearum* augustus species model was used as this is the most closely related species to *C. dispersa* available. The assembled genome completeness was evaluated using the Benchmarking Universal Single-Copy Orthologs tool, BUSCO (Simão et al. 2015). BUSCO was done on all contigs bigger than 1 Kb, using the fungal lineage dataset.

### Results and discussion

**Fig. 2:**
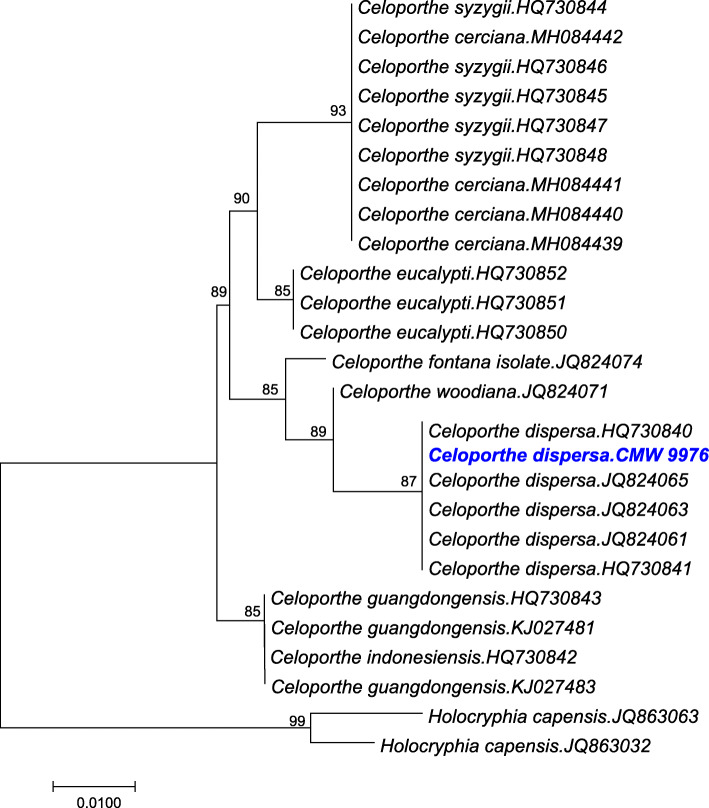
Neighbor Joining (NJ) tree of the partial gene sequences of translation elongation factor 1-α of selected reference sequences of different *Celoporthe* species. *Holocryphia capensis* was used as an outgroup in our analysis. MAFFT version 7 (Katoh and Standley 2013) was used for making alignments. The phylogenetic analysis was conducted in MEGA 7 (Tamura et al. 2013). The optimal tree with the sum of branch length = 26.62500000 is shown. The percentage of replicate trees in which the associated taxa clustered together in the bootstrap test (1000 replicates) are shown next to the branches. The *Celoporthe dispersa* CMW 9976 isolate used in this study is shown in blue and bold

Phylogenetic analysis using partial gene sequence of translation elongation factor of the sequenced genome confirmed the taxonomic identity as *C. dispersa* (Fig. 2). The assembly of *C. dispersa* consisted of 19 scaffolds, with the N50 of 1,993,378 bp. The calculated genome size was around 40 Mb and with a CG content of 52.9%. This assembly was also predicted to have 12,078 ORFs based on the gene models for *Fusarium graminearum*. Based on BUSCO analysis, this draft genome assembly had 94% completeness confirming the presence of these core eukaryotic genes. Out of this, 93% were present as single-copy genes. In our analysis, 1% of the BUSCO orthologs were found to be duplicated and 3.4% of the genes were missing. Only 39 BUSCO orthologs were classified as missing or fragmented out of the possible 1315 groups searched.

The estimated genome size and gene number for *C. dispersa* is similar to that of other species in Cryphonectriaceae, such as *Chrysoporthe austroafricana* (44.6 Mb, 13,484) (Wingfield et al. 2015a), *Chr. cubensis* (42.6 Mb, 13,121) (Wingfield et al. 2015b), *Chr. deuterocubensis* (43.9 Mb, 13,772) (Wingfield et al. 2015b) and *Cryphonectria parasitica* (43.9 Mb, 11,184) (http://genome.jgi.doe.gov/Crypa2/Crypa2.home.html). The availability of the *C. dispersa* genome from this study will enable various genomic comparisons between species of Cryphonectriaceae to be conducted. Additionally, the genome can be used to study pathogenicity, mating type and other important factors in phytopathology. The availability of the *C. dispersa* genome will enable us to include this genome within the Cryphonectriaceae during genomic comparison studies.

*Authors:* **B.D. Wingfield** * **, T.A. Duong, M. Sayari, M. A. Ferreira, A. M. Kanzi.**

*Contact: brenda.wingfield@fabi.up.ac.za

## IMA GENOME-F 12C

**Draft genome sequence of** ***Sporothrix phasma.***

### Introduction

The Ophiostomatales, defined by the single family Ophiostomatacea, accommodates 11 well-defined genera including *Sporothrix* (De Beer et al. 2016a; De Beer et al. 2016b). *Sporothrix* was recently separated from *Ophiostoma* based on analyses of sequence data for multiple gene regions (De Beer et al. 2016a). This genus now accommodates more than 50 species that reside in six species complexes and five additional groups (De Beer et al. 2016a).

Species of *Sporothrix* are found in various habitats including on wood, in soil, and in association with arthropod vectors (Roets et al. 2007; Seifert et al. 2013; Lopes-Bezerra et al. 2018). Several *Sporothrix* species cause human and animal diseases, the best-known of which is *S. shenckii* (Teixeira et al. 2014), but most are considered saprotrophs. Some species also occupy the very unusual ecological habitat within the floral heads (infructescences) of *Protea* spp., which represent an important component of the Cape Floristic Region of South Africa (Cowling and Richardson 1995; Roets et al. 2006; Roets et al. 2009b). Twelve *Sporothrix* species have been collected and described in association with *Protea* spp. (Roets et al. 2006; Roets et al. 2008; Roets et al. 2010; Ngubane et al. 2018).

*Sporothrix phasma* was described by Roets et al. (2006) where it was first discovered in the infructescence of *Protea laurifolia* and *Protea neriifolia*. This species, together with others occupying the unusual *Protea* niche, were shown to be vectored by mites and *Protea* pollinating beetles (Roets et al. 2009a). However, this system is very complex and it has recently been shown that the *S. phasma* spore-carrying mites, are phoretic on larger mites, which in turn are phoretic on *Protea*-pollinating birds (Theron-De Bruin et al. 2018). In order to better understand the processes that have allowed *Sporothrix* species to adapt to different and diverse habitats, the genome of *S. phasma* was sequenced. The broader intention was that this sequence will contribute to the basal genomic data required to study the biology, ecology and, in some cases, pathogenicity of these fungi.

### Sequenced strains

South Africa, isolated from *Protea laurifolia*, 2005, F. Roets, (culture CBS 119721 = CMW 20676 (ex- holotype); PREM 58941- dried culture).

### Nucleotide sequence accession number

The genomic sequence of *Sporothrix phasma* (CMW 20676, CBS 119721) has been deposited at DDBJ/EMBL/GenBank under the accession number WJIH00000000. The version described in this paper is version WJIH01000000.

### Materials and methods

*Sporothrix phasma* isolate CMW 20676 was obtained from the culture collection (CMW) of the Forestry and Agricultural Biotechnology Institute (FABI), the University of Pretoria, South Africa. Genomic DNA was extracted using the method described by Duong et al. (2013). Two pair-end libraries (350 bp and 550 bp average insert size) were prepared and sequenced using the Illumina HiSeq 2000 platform with 100 bp read length. Trimmomatic v. 0.38 (Bolger et al. 2014) was used for quality and adapter trimming. The program SPAdes v. 3.11.1 (Bankevich et al. 2012) was used to assemble the genome. SSPACE-standard v. 3.0 (Boetzer et al. 2011) was used for further scaffolding of SPAdes scaffolds. GapFiller v. 1–10 (Boetzer and Pirovano 2012) was used to fill or extend the assembly gaps. The Benchmarking Universal Single-Copy Orthologs (BUSCO v. 3.1.0) program (Simão et al. 2015) was used to assess the completeness of the assembly using the Sordariomyceta odb9 dataset. Maker v. 2.31.8 (Holt and Yandell 2011) was used to predict the number of protein coding genes present in the assembled genome.

The taxonomic placement of *S. phasma* in the genus *Sporothrix* was investigated by phylogenetic analysis of four combined gene regions, the Large Subunit (LSU) of the nuclear ribosomal RNA (rRNA) gene, the Internal Transcribed Spacer (ITS) regions, β tubulin (BT) gene and calmodulin (CAL) gene. The sequences of representative isolates in this genus were obtained from GenBank, as reported by De Beer et al. (2016a), and aligned online with MAFFT v. 7 (Katoh and Standley 2013). A maximum likelihood analysis was performed with the sequence data, using RaxML v. 8.2.4 (Stamatakis 2014) on the CIPRES Science Gateway v. 3.3 (Miller et al. 2010) and 1000 bootstrap replicates were performed to obtain branch support values. The genus *Ophiostoma* was used as outgroup.

### Results and discussion

**Fig. 3:**
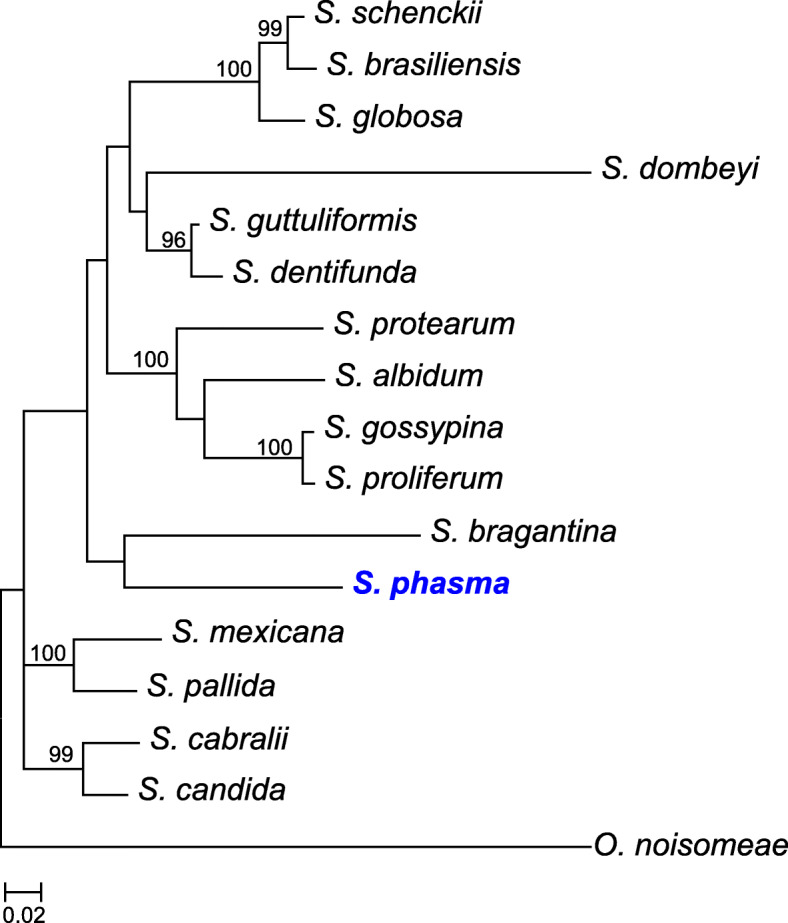
Phylogenetic tree depicting the relationship between genome sequenced *S. phasma* (in blue and bold) and related species in genus *Sporothrix*. The tree was constructed from LSU, ITS, BT and CAL gene regions using RaxML. Bootstrap support (1000 replicates) are indicated at the nodes

More than 6 million read pairs were obtained after the quality trimming. *De-novo* assembly using SPAdes resulted in 487 scaffolds which were larger than 500 bp. The number of final scaffolds was reduced to 279 after scaffolding with SSPACE and filling gaps with GapFiller. The current assembly has an N50 of 306 Kb and size of 30.2 Mb, with an overall GC content of 57.36%. The assembly included 96.8% complete, 1.2% fragmented, and 2.0% missing, BUSCOs. Maker predicted a total of 7999 protein coding genes. The taxonomic placement of *S. phasma* in *Sporothrix* is illustrated in Fig. 3. *Sporothrix phasma* has the smallest genome size when compared to other *Sporothrix* species for which genome sequences are available. *Sprorothrix pallida* has the largest genome size (37.8 Mb), followed by *S. globosa* (33.5 Mb), *S. brasiliensis* (33.2 Mb) and *S. schenckii* (32.3 Mb) (D’Alessandro et al. 2016; Huang et al. 2016; Gomez et al. 2018).

The unique habitat of *S. phasma* and its close phylogenetic relationship with various pathogenic taxa in the genus, make the data generated in this study useful for comparative genomics studies. It is intended that these will seek a better understanding of the mechanisms that underpin the ability of *Sporothrix* species to occupy a remarkable diversity of habitats. Opportunities should also arise to understand why some *Sporothrix* species have evolved to be animal and plant pathogens.

*Authors*: R. Chang, M.J. Wingfield, Z.W. De Beer, B.D. Wingfield, T.A. Duong*.

*Contact: tuan.duong@fabi.up.ac.za

## IMA GENOME-F 12D

**Metagenome sequences of a lichen species** ***Alectoria sarmentosa*** **(Parmeliaceae, Ascomycota).**

### Introduction

The advent of DNA sequencing has advanced our understanding on biodiversity research (Bik et al. 2012). The analyses of single and mutlilcous datasets in phylogenetic frameworks are frequently used for biodiversity assessment. However, these belong to a small portion of a genome and may bias the accurate species assessment. Therefore a larger portion of genome or whole genome is crucial for a more complete biodiversity assessment. More recently, genome and metagenomic data is being used for biodiversity research. For example, metagenomic data has been shown to be useful for accurate species assessment (including cryptic) especially in mixed sample or multi-lineage assemblages of organisms (‘holobionts’) such as metazoan parasites (Bass et al. 2015), viral pathogens (Bibby 2013) and other microbial communities (Louca et al. 2016).

Lichens represent iconic examples of multi-lineage assemblages of organisms. Lichens form obligate symbiotic association between fungus (exhabitant or host) and microalgae, including cyanobacteria (Hawksworth 2015). In addition to the fungal and photosynthetic partners, a wide variety of non-photosynthetic bacteria, additional fungi as enodlichenic and lichenicolous fungi are regularly found in the lichen symbiosis (Hawksworth 2015; Grube and Wedin 2016; Lumbsch and Rikkinen 2017).

The appearance of cryptic species is a common phenomenon in lichen forming fungi and fungi in general (Crespo and Lumbsch 2010; Lumbsch and Leavitt 2011; Leavitt et al. 2016; Grube et al. 2017). Cryptic biodiversity is an essential component of biodiversity that will be considerably affected by global climate change in the next decades (Bálint et al. 2011). While the molecular studies based on single and multicolus dataset have revolutionized our understanding of species delimitations in lichen-forming fungi, the application of small portion of genome may obscure the accurate cryptic biodiversity assessment.

Here we sequenced metagenome of holobiont of a lichen species *Alectoria sarmentosa* (Parmeliaceae, Ascomycota) to advance biodiversity research. Our data will also be helpful for accurate assessment of cryptic biodiversity. *Alectoria sarmentosa* is fruticose lichen belongs to the alectorioid clade of the family Parmeliaceae (Divakar et al. 2015). It is distinguished in having pendent, yellow to greenish grey thallus, pseudocyphellae fusiform, soralia rare and tuberculate (Brodo and Hawksworth 1977). The species is wide spread in Europe, Asia and North America and has been frequently used in bio-monitoring studies especially monitoring forest health (Velmala and Myllys 2011).

### Sequenced strains

*Alectoria sarmentosa*: Norway: *Telemark*: Close to Notodden village, road E134 at the bus stop called Jepertjónn, 59.36 N 09.25 W, alt., 474 m, in a sparse forest of *Pinus sylvestris* and *Picea abies*, on *Pinus sylvestris* trunk, 20 Aug. 2015, *C. G. Boluda*, [identified by C. G. Boluda] (MAF-Lich 21,536).

### Nucleotide sequence accession numbers

The *Alectoria sarmentosa* binned metagenome project has been deposited at DDBJ/ENA/GenBank under accession no. PRJNA588068.

### Materials and methods

*Alectoria sarmentosa* was collected from a bark tree in Telemak, Norway and a partial piece of thallus was deposited at the MAF Herbarium (Complutense University of Madrid, Spain) with code MAF-Lich 21,536. The sample was identified using classical procedures as morphology and chemistry; as well as molecular technique with internal transcribed spacer (ITS) sequence of rDNA.

#### Genomic DNA extraction, sequencing and trimming

Total genomic DNA of the lichen holobiont was extracted from thalli using a commercial kit *Quick*-DNA™ Fungal/Bacterial Miniprep Kit and following the manufacturers’ instruction. DNA concentration was calculated using the Qubit dsDNA dBR assy kit (Thermo Fisher Scientific, San Diego, CA). Two paired-end libraries (300 bp and 900 bp) were built using a Illumina Tru-Seq library preparation kit. Sequencing was carried out the Unidad de Genómica (Parque Científico de Madrid, Madrid, Spain) with Illumina Miseq platform (250 bp paired-end reads). Raw sequences were downloaded from Illumina BaseSpace application and were quality trimmed and filtered using Trimmomatic-0.36 (Bolger et al. 2014) with following parameters: 4 base long sliding windows, a minimun queality value of 30 and minimun length of 30 bases. (LEADING:3 TRAILING:3 SLIDINGWINDOW:4:15 MINLEN:36).

#### Genome assembly, taxonomy assignment and gene prediction

The trimmed paired-end reads were assembled using MetaSPAdes (Nurk et al. 2017) using default parameters and checking the suitability of k-mer (K21, K33, K55 and K77). In order to extract lichen-forming fungal contigs from the metagenome assembly, scaffolds of metagenome were subjected to BLASTX searches using DIAMOND (Buchfink et al. 2015) against a custom database comprising the protein sets of Archaea, Bacteria, Eukaryota, and Viruses of the NCBI nr database (downloaded in August 2018), in addition, 150 complete fungal genomes and 20 algal genomes were added from JGI. Four additional unpublished Parmeliaceae genomes generated from axenic cultures from species within Parmeliaceae [*Cetraria islandica*, *Parmelina carporrhizans*, unpublished; *Evernia prunastri* and *Pseudevernia furfuracea* (Meiser et al. 2017)] were used as reference genomes for taxonomy assignment, taking only scaffolds belonging to Parmeliaceae. The results of the DIAMOND search were then used as input for MEGAN6 (Huson et al. 2016) for taxonomic assignment (parameters: min-support = 1, min-score = 50, top-hit = 10%, no low complexity filtering).

Contigs belonging to Parmeliaceae were extracted and completeness of *Alectoria sarmentosa* genome was evaluated with QUAST (Gurevich et al. 2013) and BUSCO approach using Pezizomycotina dataset (Simao et al. 2015). Gene prediction was conducted with MAKER2 (Holt and Yandell 2011), using Augustus v. 3.22 (Stanke et al. 2006) and GeneMarkES (Ter-Hovhannisyan et al. 2008). In order to identify biosynthetic gene clusters, AntiSMASH pipeline v. 4 (Blin et al. 2017) was conducted on contigs with default parameters.

#### Phylogeny

The complete internal transcribed species region ((ITS1, 5.8S, ITS2; ~ 500 bp), commonly used for species delimitation in Parmeliaceae and the standard DNA barcode for fungi (Schoch et al. 2012) was extracted from *Alectoria sarmentosa* genome sequence. This was aligned with other ITS sequences of *Alectoria* species downloaded from NCBI (https://www.ncbi.nlm.nih.gov/). Sequences were aligned using the program MAFFT v. 7 (Katoh and Standley 2013) and the program Gblocks v. 0.91b (Talavera and Castresana 2007) was used to delimit and remove ambiguous alignment nucleotide positions. Maximum likelihood analysis (ML) was conducted with RAxML v. 8.1.11 (Stamatakis 2014), using CIPRES Science Gateway server (http://www.phylo.org/portal2/). Nodal support was evaluated with 1000 bootstrap pseudoreplicates. Phylogenetic trees were drawn using the program FigTree v. 1.4.2 (Rambaut 2009).

### Results and discussion

The metassembly of holobiont resulted in 137,274 scaffolds and 161,465,382 bp of length. After taxonomic assignment, we consider all assigned contigs to Parmeliacea belong to *Alectoria sarmentosa* and yielded the draft genome of 46,540,876 bp (46.5 Mb) assembled into 1788 contigs. Of these, 1046 were longer than 1000 bp and contained 46,193,838 bp (46.1 Mb) of genome. The largest contig was 400,628 bp and the N50 and L50 were 92,863 bp and 140 bp, respectively (Table 2). The genome had a GC content of 40.34% and average coverage of assembly was 53.97. The assessment of genome completeness of our draft genome assembly based on 3156 single-copy orthologos BUSCO genes showed that most of the gene space was covered (96.3%). *A. sarmentosa* draft genome assembly contained 3041 complete and single-copy BUSCOs, 35 fragmented, 26 duplicated and 80 missing BUSCO genes out of the 3156 BUSCO genes searched (Table 2; Simao et al. 2015).

**Table 2:** Assembly metric, genome completeness and number of biosynthetic gene clusters (BGC) of *Alectoria sarmentosa* binned metagenome

Assembly Metrics		Genome Completeness		Biosynthetic Gene Clusters	
Total length	46,540,876 bp	Completeness	96.3%	PKS Type I	13
Number of contigs	1788	Complete (C)	3041	PKS Type I-NRPS	5
Number of contigs (>1000 bp)	1046	Single-Copy (SC)	3015	PKS Type III	1
GC content	40.34%	Duplicated (D)	26	PKS Type III -NRPS	1
N50 contig length	92,863 bp	Fragmented (F)	35	NRPS	2
Number of genes	9695	Missing (M)	80	INDOLE	2
				TERPENE	2
				OTHER	4

**Fig. 4:**
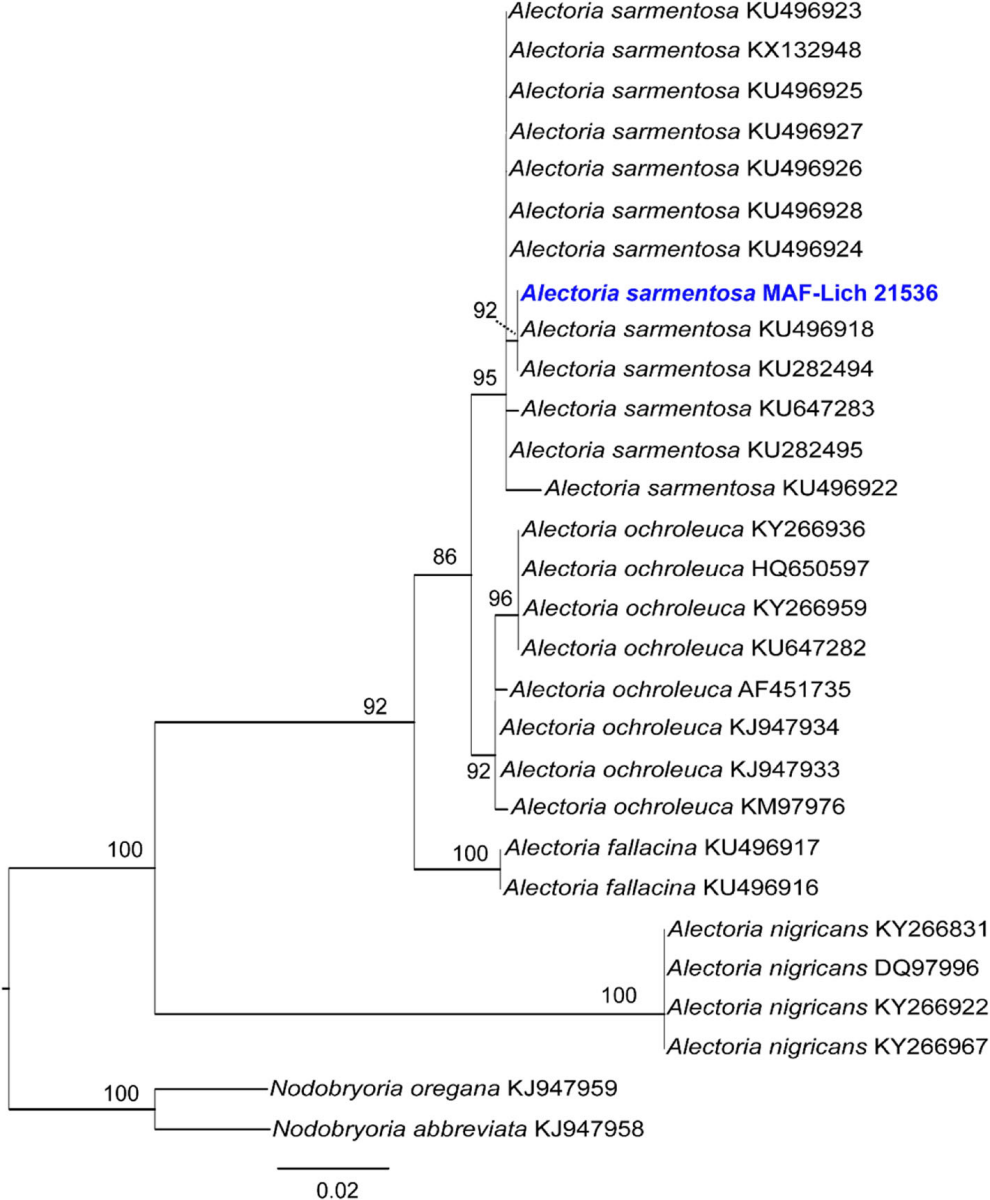
A maximum likelihood (ML) tree based on ITS sequences of *Alectoria* species including the genome sequence of *Alectoria sarmentosa* reported here. The ML tree obtained with RAxML is shown. The isolate from which the genome was sequenced is indicated in blue and bold. ML bootstrap values ≥75% are indicated at the branches. Two species of *Nodobryoria* were used as out-group (Divakar et al. 2015)

The gene prediction conducted by MAKER2 (Holt and Yandell 2011) yielded a total of 9695 protein-coding genes (Table 2). Our results are concordant to other recently sequenced genomes of lichen-forming fungi (e.g., *Caloplaca flavorubescens*: 9695 genes; Park et al. 2013a. *Cladonia macilenta*: 7322; Park et al. 2013b. *Endocarpon pusillum*: 9285; Wang et al. 2014. *Ramalina intermedia*: 8871; Wang et al. 2018b). The analysis of AntiSMASH (Blin et al. 2017) resulted in 61 metabolic gene clusters. The metagenome sequence of *A. sarmentosa* reported here is the first published genome sequence of the alectorioid clade (Table 2; Fig. 4). The alectorioid clade includes *c.* Seventy described species distributed in five genera viz.: *Alectoria*, *Bryoria*, *Bryocaulon*, *Nodobryoria*, and *Pseudephebe* (Divakar et al. 2015). Phylogenetic analysis of ITS sequence from the sequenced genome confirmed the taxonomic identity as *A. sarmentosa* (Fig. 4). The draft genome of *A. sarmentosa* generated in this study will add to the already growing genome database of lichen forming-fungi for future studies of evolutionary biology like speciation or cryptic species discovery, as well as comparative genomic or biosynthetic gene clusters studies. Furthermore, the availability of a genome sequence also provides the opportunity to develop molecular markers, for example species-specific single nucleotide polymorphism (SNPs) markers, or mating types (Alors et al. 2017), which would be important for population studies of this and other closely related taxa.

*Authors:* **D. Pizarro, A. Crespo, P.K. Divakar** *.

*Contact: pdivakar@farm.ucm.es

## Data Availability

All data and material are available the relevant details (data banks, culture collections and herbaria) are given in the manuscript.
